# Revealing clinically relevant specific IgE sensitization patterns in Hymenoptera venom allergy with dimension reduction and clustering

**DOI:** 10.1016/j.waojou.2023.100820

**Published:** 2023-09-30

**Authors:** Robert Kaczmarczyk, Tobias Lasser, Tilo Biedermann, Johannes Ring, Alexander Zink

**Affiliations:** a Technical University of Munich, School of Medicine, Department of Dermatology and Allergy, Munich, Germany; b Technical University of Munich, School of Computation, Information and Technology, Department of Informatics, Munich Institute of Biomedical Engineering, Munich, Germany; c Division of Dermatology and Venereology, Department of Medicine Solna, Karolinska Institutet, 17176, Stockholm, Sweden

**Keywords:** Hymenoptera venom allergy, Specific IgE, Risk assessment, Cohort-study

## Abstract

**Background:**

Immunoglobulin E (IgE) blood tests are used to detect sensitizations and potential allergies. Recent studies suggest that specific IgE sensitization patterns due to molecular interactions affect an individual's risk of developing allergic symptoms.

**Objective:**

The aim of this study was to reveal specific IgE sensitization patterns and investigate their clinical implications in Hymenoptera venom allergy.

**Methods:**

In this cross-sectional study, 257 hunters or fishers with self-filled surveys on previous Hymenoptera stings were analyzed. Blood samples were taken to determine Hymenoptera IgE sensitization levels. Using dimensionality reduction and clustering, specific IgE for 10 Hymenoptera venom allergens were evaluated for clinical relevance.

**Results:**

Three clusters were unmasked using novel dimensionality reduction and clustering methods solely based on specific IgE levels to Hymenoptera venom allergens. These clusters show different characteristics regarding previous systemic reactions to Hymenoptera stings.

**Conclusion:**

Our study was able to unmask non-linear sensitization patterns for specific IgE tests in Hymenoptera venom allergy. We were able to derive risk clusters for anaphylactic reactions following hymenoptera stings and pinpoint relevant allergens (rApi m 10, rVes v 1, whole bee, and wasp venom) for clustering.

**Key messages**

• UMAP dimension reduction and HDBSCAN clustering can be used synergically to derive clusters from quantitative specific IgE sensitization levels to Hymenoptera venom allergens
• Cluster association has clinical implications on previous systemic reactions to Hymenoptera stings
• We suggest a more resource-efficient use of Hymenoptera specific IgE *in-vitro* tests (rApi m10, rVes v1, whole honey bee, and wasp venom)

**Capsule summary**

Anaphylactic risk assessment in Hymenoptera venom allergy could potentially benefit from the analysis of quantitative specific IgE levels using dimension reduction and clustering algorithms.

## Introduction

Allergic diseases affect as much as 30% of world's population and represent a major socio-economic burden.^1^^,^^2^ Despite low mortality, allergies may lead to significant reduction in quality of life.^3^ Symptoms range from persistent nasal obstruction and eye watering, as seen in tree pollen allergies, to severe anaphylactic reactions and death, as observed in peanut, Hymenoptera venom, and drug allergies. According to recent studies, the prevalence of allergic diseases is globally^4^^,^^5^ on the rise.

Diagnostics for IgE-mediated allergies, like Hymenoptera venom allergies, involve medical history assessment, skin tests, and *in-vitro* blood tests such as specific IgE measurements.^6^ Yet, the quantitative interpretation of specific IgE levels and their clinical relevance remains an area of contention.^7^ The current cut-off values for determining sensitivity are widely debated, and while some progress has been made in certain allergy diagnostics,^8^^,^^9^ there remain challenges in others like drug hypersensitivity^10^ and specific food allergies.^11^ Emerging research is investigating the use of IgE-ratios to assess risks of different specific allergens.^12^, ^13^, ^14^, ^15^, ^16^ These studies suggest, if at all, a complex, non-strictly linear interaction pattern of specific IgE levels and indicate varied results across allergic diagnostics.

Cross-reactive carbohydrate determinants (CCDs) present in venom extracts can complicate the identification of primary Hymenoptera allergies. To address this, molecular or component-resolved diagnosis (CRD)^17^^,^^18^ enable the production of recombinant allergen proteins free of CCDs. For Hymenoptera venom allergies, CRD isolates specific allergens, including Api m 2, 3, 4, 10, Ves v 1, and Ves v 5, establishing them as markers for primary honey bee or wasp venom allergies. In contrast, the cross-reactivity between Api m 5 and Ves v 3 hinders their use as reliable marker allergens. Distinguishing primary allergies from cross-reactivity is crucial in selecting the appropriate immunotherapy. In addition, a dominant Api m 10 sensitization is a marker for increased risk of immunotherapy failure.^19^

New evolving mathematical models, such as UMAP (Uniform Manifold Approximation and Projection),^20^ are revolutionizing the field by offering more accurate, lower-dimensional representations of higher-dimensional datasets. This stands in contrast to traditional network analysis, which often relies on less precise layouts.^21^ Specifically, UMAP is gaining traction due to its capability to better preserve both local and global structures within high dimensional data, offering advantages over traditional methods like t-Distributed Stochastic Neighbor Embedding (t-SNE)^20^ or principal component analysis (PCA).^22^

On the other hand, HDBSCAN (Hierarchical Density-Based Spatial Clustering of Applications with Noise)^23^ stands out as an advanced clustering algorithm. Unlike traditional clustering methods (e.g., k-means clustering or latent class analysis^24^) that may require specifying the number of clusters beforehand, HDBSCAN operates by identifying clusters of varying densities. When UMAP and HDBSCAN are combined, they offer an effective approach to identify and visualize clusters in reduced-dimensional space.

In this study, we applied current scalable, state-of-the-art mathematical methods to allergology by combining a dimension reduction method with a clustering algorithm to assess a) if Hymenoptera specific IgE sensitization patterns are unmasking visually in the lower dimensional space, b) the implications for laboratory measurements and clinical application of these methods, and c) if these methods can help to optimize the allocation of resources and suggest a more cost-efficient use of laboratory tests.

## Methods

### Study design

In a cross-sectional study conducted in the Greater Munich Area, Southern Germany, during winter 2016 and January 2017, 257 adult individuals were recruited and analyzed from annual winter meetings of three hunting associations and an international hunting and fishing exhibition.^25^ Upon providing written informed consent prior to study inclusion, participants filled out a self-reported questionnaire capturing general demographics, hunting/fishing activities, known allergies, and experiences with Hymenoptera stings. The questionnaire assessed reactions to insect stings based on local reactions and questions according to the European Grading of Anaphylactic Symptoms^26^^,^^27^ (anaphylaxis grade 1 to grade 4). Blood samples were subsequently collected and stored for later *in vitro* testing. The ImmunoCAP™ system measured total and specific IgE levels, along with serum tryptase.^25^

### Variables, data sources, measurement

Our primary independent variables were the subject age, total IgE (kU/l), tryptase (μg/l), and each subject's individual subset of measured specific IgE levels (kUA/l) to 10 different Hymenoptera allergens (rApi m 1, rApi m 2, rApi m 3, rApi m 5, rApi m 10, rVes v 1, rVes v 5, and MUXF3, a CCD marker molecule) using ImmunoCAP™ Allergy Testing Solutions and Phadia™ Laboratory System by Thermo Fisher Scientific.

### Dimension reduction

**Fig. 1:**
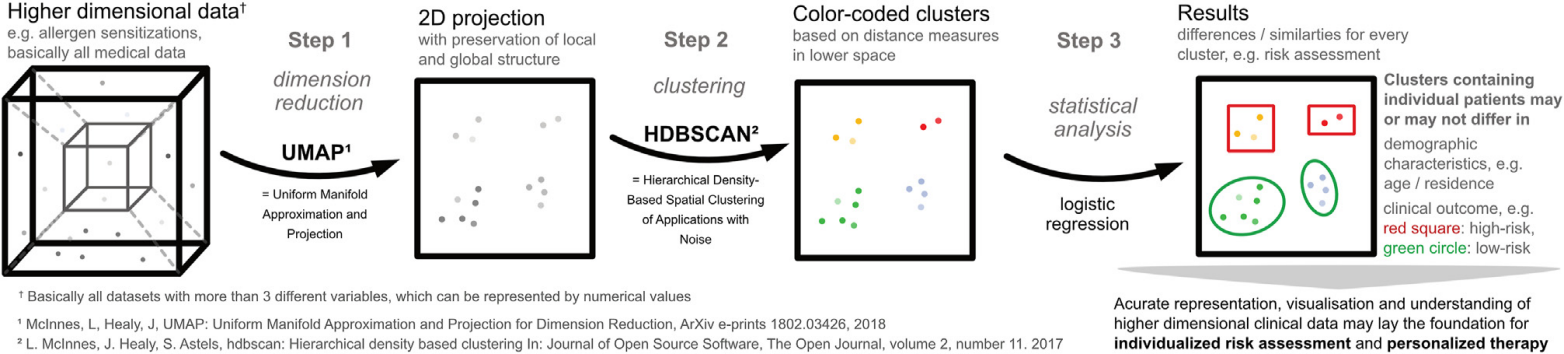
Dimension reduction and clustering algorithm. A step-by-step study protocol for clustering higher dimensional data after applying dimensionality reduction

Dimension reduction was solely performed using specific IgE values. Uniform Manifold Approximation and Projection (UMAP)^28^ was performed (Fig. 1). The result was a two-dimensional representation of the higher dimensional specific IgE measurements with preserved local and global structure.^20^

### Clustering

**Fig. 2:**
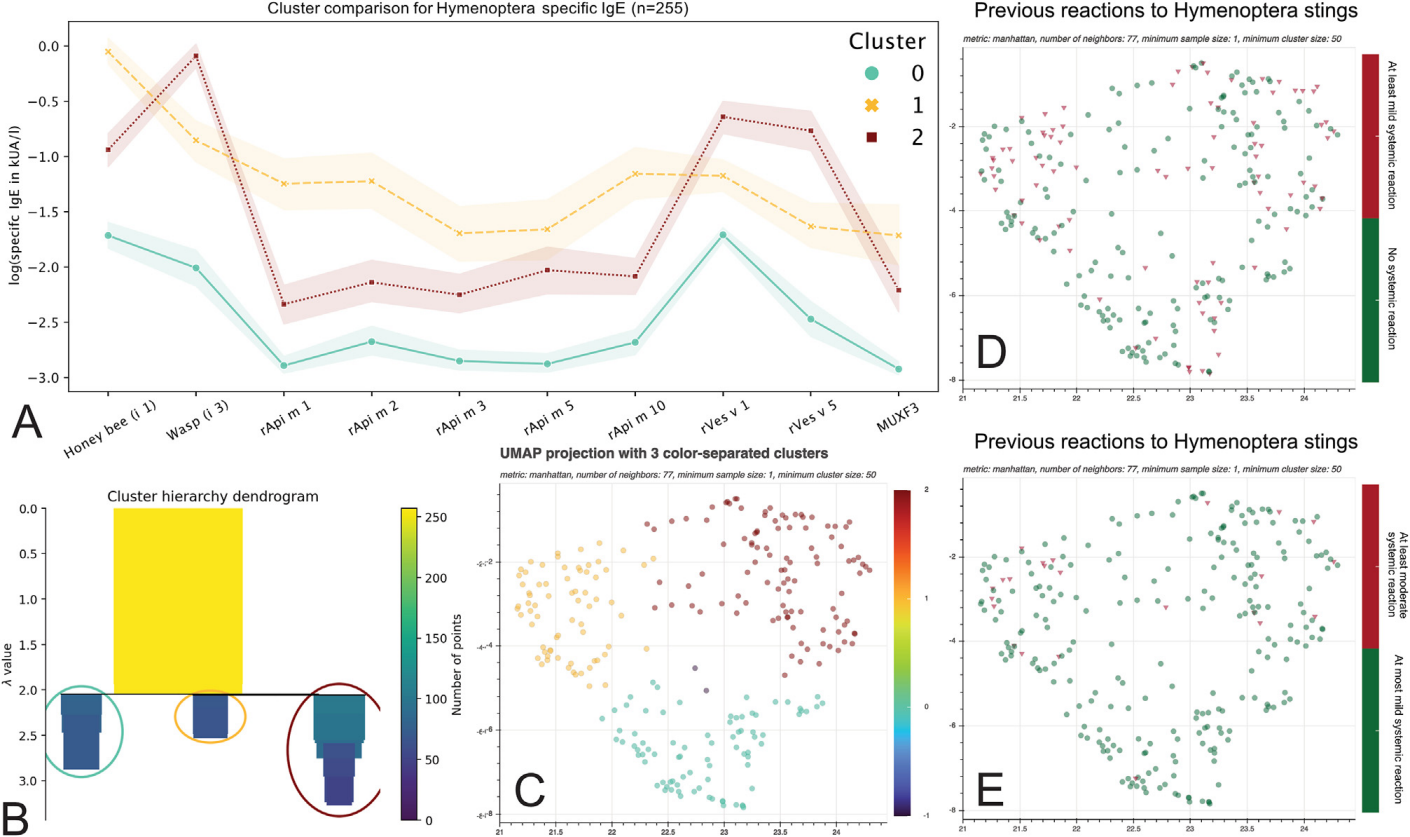
Cluster analysis through specific IgE sensitization patterns to Hymenoptera venom allergens (n = 257). Mean specific IgE values with 95% confidence intervals for the different Hymenoptera venom allergens in the three different clusters in the hunter/fisher group (A). Two unclustered subjects were omitted. Clustering parameters were chosen with the help of the cluster hierarchy dendrogram (B). The lower dimensional representations, also called embeddings, are visualized and color-coded by associated cluster (C), a subject's previous systemic reaction, (D) and a subject's previous moderate to severe systemic reaction following Hymenoptera stings (E)

**Fig. 3:**
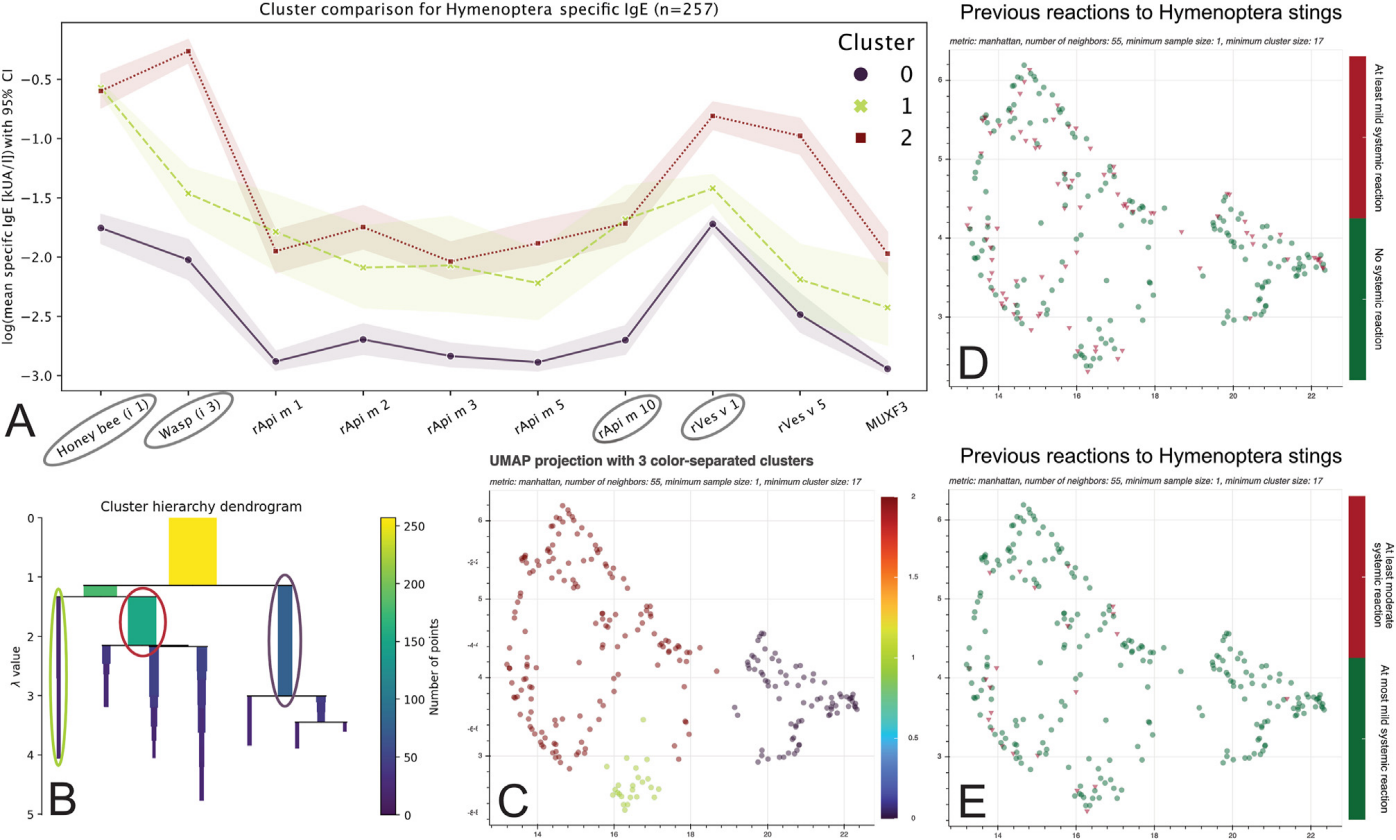
Cluster analysis through specific IgE sensitization patterns to Hymenoptera venom allergens (n = 257). Mean specific IgE values with 95% confidence intervals for the different Hymenoptera venom allergens in the three different clusters in the hunter/fisher group. Only both whole venom extracts honey bee (i 1), wasp (i 3), and the recombinants rVes v 1 and rApi m 10 (circled) were used in the dimension reduction and clustering analysis (A). Clustering parameters were chosen with the help of the cluster hierarchy dendrogram (B). The lower dimensional representations, also called embeddings, are visualized and color-coded by associated cluster (C), a subject's previous systemic reaction, (D) and a subject's previous moderate to severe systemic reaction following Hymenoptera stings (E)

A high-performance implementation^23^ of Hierarchical Density-Based Spatial Clustering of Applications with Noise (HDBSCAN)^29^ was utilized, using unsupervised learning to find clusters (= dense regions) in the embeddings (Fig. 1). After visual exploration of the lower-dimensional representation and the cluster hierarchy dendrogram (Fig. 2+3, B), we set the parameters to reach the best fit of the projected dense regions. We used *Manhattan* as a distance metric for higher-dimensional.^30^ A dot in the graph represents an individual subject. The subjects are either colored by cluster association or other attributes as stated (Fig. 2, Fig. 3). For reproducibility, a random seed of 42 was set.

### Statistical analysis

Data were analyzed using Python 3.8.8 with statistical libraries pandas 1.3.0, SciPy 1.5.2, statsmodels 0.12.0, umap 0.5.1 and hdbscan 0.8.26. In addition to descriptive parameters, we report all results as mean with 95% confidence intervals. Specific IgE graphs were log-transformed for better comparability. Logistic regression was performed for binary outcome variables using Broyden-Fletcher-Goldfarb-Shanno algorithm (BFGS) as an optimizer, and odds ratios with 95% confidence intervals and p-values are shown.

## Results

### Cluster analysis of the hunter/Fisher group

UMAP dimension reduction followed by HDBSCAN with the help of the cluster hierarchy dendrogram revealed 3 principal clusters derived from specific IgE sensitizations to Hymenoptera venom allergens in the hunter/fisher group (Fig. 2, A-C). Subjects not affiliated with any of the 3 clusters (n = 2, 0.78%) or who did not answer the question on previous Hymenoptera stings (n = 12, 4.7%) were excluded from the further analysis.

### Age, tryptase, total IgE, and previous stings in different clusters

The age and tryptase levels of the subjects in the respective 3 clusters did not differ. The total IgE was significantly lower in cluster 0 (46.2 ± 15.8) compared to cluster 2 (164.9 ± 67.6, p = 0.005), whereas both clusters were indistinguishable from cluster 1 (128.5 ± 54.0, p = 0.11 and p = 0.61). Regarding previous stings, cluster 1 (85.5% ± 8.5) had significantly more subjects with more than 5 stings than cluster 0 (56.4% ± 11.3, p = 0.001) and cluster 2 (61.4% ± 9.9, p = 0.003), while the latter 2 clusters did not show any significant difference (p = 0.74).

### Specific IgE characteristics of whole venom extracts

The analysis of whole venom extracts for honey bee (i 1) and wasp (i 3) revealed a different focus for every cluster. While specific IgE for honey bee (i 1) was only different in clusters 0 and 1 (mean difference: 2.23 ± 3.13, p = 0.003), wasp (i 3) was significantly different between clusters 0 and 2 (mean difference: 2.22 ± 2.18, p = 0.001) as well as between clusters 1 and 2 (mean difference: 1.78 ± 2.26, p = 0.001).

### Specific IgE characteristics of Hymenoptera recombinants

Specific IgE characteristics of Hymenoptera recombinants were analyzed across 3 distinct clusters. The variations in specific IgE levels between clusters for the tested recombinants are summarized in the table below (Table 1). Specific IgE values for r Api m 1, 2, 3, and 10 where highest in cluster 1 compared to the other 2 clusters. In contrast, the specific IgE values for r Ves v 1 and 5 had their peak values in cluster 2. Notably, rApi m 5 and CCD MUXF3 showed no significant variations in specific IgE levels across the clusters. When considering the mean specific IgE values for all recombinants, values were notably lower in cluster 0 compared to clusters 1 and 2, with no significant difference between clusters 1 and 2.

**Table 1:** Comparative IgE Sensitization across clusters. The table displays the mean and 95% confidence intervals of specific IgE levels for various Hymenoptera venom recombinants across three distinct clusters (Cluster 0, 1, and 2). Significant differences between the clusters with their respective p values are derived from a Tukey's HSD test after a one-way ANOVA. Allergens with no significant variations across clusters (p > 0.05) are shaded in grey. Specific IgE levels for a given cluster that demonstrates higher levels compared to the other two clusters are emphasized in bold

Allergen	Cluster 0 (Mean ± 95% CI)	Cluster 1 (Mean ± 95% CI)	Cluster 2 (Mean ± 95% CI)	Differences	P values
rApi m 1	0.0019 ± 0.0016	**0.57 ± 0.46**	0.039 ± 0.03	C1 > C0C1 > C2	0.004 0.05
rApi m 2	0.008 ± 0.005	**0.73 ± 0.69**	0.11 ± 0.1	C1 > C0C1 > C2	0.018 0.037
rApi m 3	0.004 ± 0.003	**0.14 ± 0.07**	0.05 ± 0.04	C1 > C0C1 > C2	0.001 0.036
rApi m 5	0.003 ± 0.003	0.26 ± 0.17	0.57 ± 0.84		
rApi m 10	0.005 ± 0.003	**0.76 ± 0.85**	0.048 ± 0.03	C1 > C0C1 > C2	0.045 0.049
rVes v 1	0.025 ± 0.005	0.23 ± 0.17	**0.96 ± 0.43**	C2 > C0C2 > C1	0.001 0.003
rVes v 5	0.013 ± 0.005	0.097 ± 0.04	**0.93 ± 0.54**	C2 > C0C2 > C1	0.0013 0.006
CCD MUXF3	0.0009 ± 0.0007	0.22 ± 0.14	0.41 ± 0.47		
Allergen Avg.	0.012 ± 0.002	0.57 ± 0.38	0.62 ± 0.32	C1 > C0C2 > C0	0.026 0.006

In summary, cluster 0 was characterized by peaks in specific IgE for honey bee (i 1), the wasp recombinant rVes v 1, and low levels of CCD MUXF3. Cluster 1 showed the highest average specific IgE to all tested allergens, with a peak in specific IgE for honey bee (i 1). Cluster 2 primarily consisted of high peaks in specific IgE for whole wasp venom (i 3) followed by the wasp recombinants rVes v 1 and rVes v 5 (Fig. 2, A).

### Clinical manifestation of Hymenoptera venom allergies

In the previous section, we elaborated on the implications of cluster affiliations on Hymenoptera specific IgE sensitization patterns. When cluster association on previous systemic reactions following Hymenoptera stings was evaluated, we found an increasing risk from cluster 0, (23.1%) to cluster 2 (38.5%), and to cluster 1 (42.0%, OR: 0.56 [0.38, 0.83], p = 0.003). A medical history of more than 5 previous Hymenoptera stings had non-significant implications on previous systemic reactions to Hymenoptera stings (40.1% vs 23.5%, OR: 1.53 [0.95, 2.47], p = 0.08). Higher specific IgE levels in subjects who had systemic reactions following Hymenoptera stings were seen for rApi m 10 (OR: 7.29 [27.28, 1.95], p = 0.003) and rApi m 1 (OR: 5.29 [1.25, 22.29], p = 0.023), while the whole honey bee venom (i 1) extract showed an opposite trend (OR: 0.23 [0.07, 0.74], p = 0.013). All other Hymenoptera specific IgE levels (wasp (i 3), rApi m 2, 3, 5, rVes v 1, 5 and MUXF3) showed no predictive benefit. In the case of at least moderate systemic reactions to Hymenoptera stings, the only predictor was the cluster association (OR: 0.17 [0.09, 0.32], p < 0.001, Table 2, Fig. 2), which is reflected in the increased number of at least moderate systemic reactions from the lower-risk cluster 0 (1.3%) over the intermediate risk cluster 2 (9.4%) to the highest-risk cluster 1 (17.4%).

**Table 2:** Binary logistic regression for anaphylactic reactions in the hunter/fisher group. Results of the binary logistic regression model of the given independent variables (more than 5 stings, cluster association and specific IgE levels to Hymenoptera venom recombinants) as predictors for (A) at least a mild systemic reaction or (B) at least a moderate systemic reaction following a Hymenoptera sting. Clusters were derived from the complete Hymenoptera panel. Cluster association as well was the levels of honey bee (i 1), rApi m 1 and rApi m 10 specific IgE sensitization showed a predictive value for systemic reactions to Hymenoptera stings. For moderate to severe systemic reactions following Hymenoptera stings, cluster association was the only variable which provided a predictive value in our model

Variables	coef	[0.025	0.975]	std err	z	Odds Ratio	[0.025	0.975]	P>|z|	Sig.
**(A) At least mild systemic reaction**										
>5 Stings	0.4272	−0.051	0.906	0.244	1.75	1.533	0.95	2.474	0.08	n.s.
**Cluster association**	−0.7263	−1.021	−0.432	0.15	−4.832	**0.484**	**0.36**	**0.649**	<0.001	∗∗∗
**Honey bee (i 1)**	−1.4547	−2.607	−0.302	0.588	−2.474	**0.233**	**0.074**	**0.739**	0.013	∗
Wasp (i 3)	0.5962	−0.053	1.246	0.331	1.799	1.815	0.948	3.476	0.072	n.s.
**rApi m 1**	1.6651	0.226	3.104	0.734	2.268	**5.286**	**1.254**	**22.287**	0.023	∗
rApi m 2	−0.007	−0.806	0.792	0.408	−0.017	0.993	0.447	2.208	0.986	n.s.
rApi m 3	0.0059	−2.089	2.101	1.069	0.005	1.006	0.124	8.174	0.996	n.s.
rApi m 5	−0.3241	−1.228	0.58	0.461	−0.703	0.723	0.293	1.786	0.482	n.s.
**rApi m 10**	1.9867	0.667	3.306	0.673	2.952	**7.291**	**1.948**	**27.276**	0.003	∗∗
rVes v 1	−0.6577	−1.399	0.084	0.378	−1.738	0.518	0.247	1.088	0.082	n.s.
rVes v 5	−0.1461	−0.657	0.364	0.26	−0.561	0.864	0.518	1.439	0.575	n.s.
MUXF3	0.9917	−0.311	2.294	0.665	1.492	2.696	0.733	9.915	0.136	n.s.
**(B) At least moderate systemic reaction**										
>5 Stings	−0.6893	−1.412	0.033	0.369	−1.869	0.502	0.244	1.034	0.062	n.s.
**Cluster association**	−1.7922	−2.458	−1.126	0.34	−5.275	**0.167**	**0.086**	**0.324**	<0.001	∗∗∗
Honey bee (i 1)	−0.1409	−1.861	1.579	0.877	−0.161	0.869	0.156	4.85	0.872	n.s.
Wasp (i 3)	−0.0335	−1.295	1.228	0.644	−0.052	0.967	0.274	3.414	0.958	n.s.
rApi m 1	0.3481	−1.4	2.096	0.892	0.39	1.416	0.247	8.134	0.696	n.s.
rApi m 2	−1.4441	−4.202	1.314	1.407	−1.026	0.236	0.015	3.721	0.305	n.s.
rApi m 3	−2.809	−8.81	3.192	3.062	−0.917	0.06	0	24.337	0.359	n.s.
rApi m 5	0.003	−1.216	1.222	0.622	0.005	1.003	0.296	3.394	0.996	n.s.
rApi m 10	0.5214	−1.922	2.964	1.246	0.418	1.684	0.146	19.375	0.676	n.s.
rVes v 1	−1.7418	−4.606	1.122	1.461	−1.192	0.175	0.01	3.071	0.233	n.s.
rVes v 5	0.571	−0.549	1.691	0.572	0.999	1.77	0.578	5.425	0.318	n.s.
MUXF3	0.5332	−1.383	2.45	0.978	0.545	1.704	0.251	11.588	0.586	n.s.

These data suggest that clusters derived from quantitative Hymenoptera-specific IgE levels are not only a predictor, among others, for previous systemic reactions but also the only predictor for previous moderate to severe systemic reactions.

### Lower-dimensional recreation of the risk-associated clusters

Next, we assessed the clusters derived only from the specific IgE sensitization levels to whole bee (i 1) and wasp (i 3) venom, rApi m 10, and rVes v 1 (Fig. 3A–C). As in the previous clusters, we observed an increasing number of patients with at least mild systemic reactions from cluster 0 (23.0%), to cluster 1 (29.2%), and to cluster 2 (40.8%, OR: 0.52 [0.38, 0.70], p < 0.001). For the prediction of moderate to severe systemic reactions, cluster association was again the only predictor (OR: 0.20 [0.11, 0.36], p < 0.001, Fig. 3, Table 3) in the logistic regression model, with an increasing risk from cluster 0 (1.4%), to cluster 2 (12.2%), and to cluster 1 (12.5%).

**Table 3:** Binary logistic regression for anaphylactic reactions in the hunter/fisher group. Results of the binary logistic regression model of the given independent variables (more than 5 stings, cluster association and specific IgE levels to Hymenoptera venom recombinants) as predictors for (A) at least a mild systemic reaction or (B) at least a moderate systemic reaction following a Hymenoptera sting. Clusters were derived from the whole venom extracts as well as rVes v 1 and rApi m 10. Both cluster association and rApi m 10 sensitization levels showed a predictive value for a systemic reaction following Hymenoptera stings. For moderate to severe systemic reactions, cluster association was the only variable providing a predictive value in our model

Variables	coef	[0.025	0.975]	std err	z	Odds Ratio	[0.025	0.975]	P>|z|	Sig.
**(A) At least mild systemic reaction**										
>5 Stings	0.4154	−0.084	0.915	0.255	1.631	1.515	0.919	2.497	0.103	n.s.
**Cluster association**	−0.6581	−0.961	−0.355	0.155	−4.258	**0.518**	**0.383**	**0.701**	<0.001	∗∗∗
Honey bee (i 1)	−0.9708	−2.076	0.134	0.564	−1.722	0.379	0.125	1.143	0.085	n.s.
Wasp (i 3)	0.4581	−0.168	1.084	0.319	1.434	1.581	0.845	2.956	0.152	n.s.
rApi m 1	1.2487	−0.178	2.675	0.728	1.715	3.486	0.837	14.512	0.086	n.s.
rApi m 2	−0.0991	−0.981	0.783	0.45	−0.22	0.906	0.375	2.188	0.826	n.s.
rApi m 3	−0.0402	−2.122	2.041	1.062	−0.038	0.961	0.12	7.698	0.97	n.s.
rApi m 5	−0.2265	−1.041	0.588	0.415	−0.545	0.797	0.353	1.8	0.586	n.s.
**rApi m 10**	1.4593	0.186	2.732	0.65	2.247	**4.303**	**1.204**	**15.364**	0.025	∗
rVes v 1	−0.5492	−1.267	0.169	0.366	−1.499	0.577	0.282	1.184	0.134	n.s.
rVes v 5	−0.0813	−0.57	0.408	0.249	−0.326	0.922	0.566	1.504	0.745	n.s.
MUXF3	0.5708	−0.69	1.831	0.643	0.888	1.77	0.502	6.24	0.375	n.s.
**(B) At least moderate systemic reaction**										
>5 Stings	−0.4433	−1.174	0.287	0.373	−1.19	0.642	0.309	1.332	0.234	n.s.
**Cluster association**	−1.6006	−2.168	−1.033	0.29	−5.525	**0.202**	**0.114**	**0.356**	<0.001	∗∗∗
Honey bee (i 1)	0.84	−0.786	2.466	0.83	1.012	2.316	0.456	11.775	0.311	n.s.
Wasp (i 3)	−0.5057	−1.875	0.864	0.699	−0.724	0.603	0.153	2.373	0.469	n.s.
rApi m 1	−0.6728	−2.222	0.877	0.791	−0.851	0.51	0.108	2.404	0.395	n.s.
rApi m 2	−1.8179	−4.864	1.229	1.554	−1.169	0.162	0.008	3.418	0.242	n.s.
rApi m 3	−4.7224	−11.45	2.003	3.431	−1.376	0.009	0	7.411	0.169	n.s.
rApi m 5	0.3974	−0.7	1.495	0.56	0.71	1.488	0.497	4.459	0.478	n.s.
rApi m 10	0.3016	−2.257	2.861	1.306	0.231	1.352	0.105	17.479	0.817	n.s.
rVes v 1	−1.8105	−4.851	1.23	1.551	−1.167	0.164	0.008	3.421	0.243	n.s.
rVes v 5	0.8726	−0.333	2.078	0.615	1.419	2.393	0.717	7.988	0.156	n.s.
MUXF3	0.0603	−1.728	1.849	0.913	0.066	1.062	0.178	6.353	0.947	n.s.

## Discussion

The role of quantitative specific IgE *in* *vitro* tests on allergy clinical manifestations has been a matter of prolonged, scientific debate.^31^^,^^32^ The findings in our study are in agreement with previous studies on the mixed importance of Hymenoptera recombinants.^33^^,^^34^ However, our study for the first time describes the introduction of dimensionality reduction paired with clustering algorithms to derive risk clusters, which have meaningful implications on the clinical characteristics of Hymenoptera venom allergies. Moreover, we were able to demonstrate how to reduce the number of necessary recombinants by evaluating specific IgE sensitization patterns in associated clusters.

More accurate dimensionality reduction methods like UMAP^28^ have already been successfully applied to medical science and used for identifying cell types and trajectories in embryology^35^ as well as the exploration of finer structures in cellular development.^36^ Novel clustering algorithms, like the recently developed HDBSCAN, implemented as both a hierarchal and density-based clustering algorithm^23^ has previously only been observed outside of the medical field, e.g., in chemistry to uncover large-scale conformational change in molecules.^37^ Furthermore, training machine learning algorithms to tackle the complex nature of specific IgE sensitization usually requires large amounts of data, whereas dimensionality reduction approaches can provide deterministic predictions on a far smaller scale without overfitting. The assessment of sensitization patterns with a different approach like *latent class analysis* (LCA) has successfully been conducted in asthmatic patients.^38^ As seen with the *k-nearest neighbors method* (KNN) for clustering, a downside of LCA though is the number of groups that need to be determined before analysis and that the dependent variables need to be categorical. The methodology around network analysis revealed previously unknown disease entities in the field of psychopathology^39^ as well as disease characteristics in asthmatic patients following sensitization clustering.^40^^,^^41^ Unlike UMAP, however, network analysis commonly uses force directed layouts,^42^ which distort the true nature of higher dimensional datasets.^21^

The risk assessment of more than 5 previous Hymenoptera stings shown in the previous study on the hunter/fisher cohort was canceled out by the higher predictive value of the cluster association in our model. The authors of the previous study were “unable to find a correlation between reaction severity and sensitization to any of the hitherto available recombinant allergens”.^25^ Our study suggests the necessity to assess the specific IgE levels in a non-linear wholesome approach. By using UMAP dimensionality reduction and HDBSCAN clustering as well as by including all Hymenoptera venom allergens quantitatively, cluster associations, and number of previous stings in a single logistic regression model with a Bayesian optimization method, we were able to show that the associated clusters are the only means to predict previous moderate to severe systemic reactions. We also demonstrated a distinction between subjects with and without systemic reactions following Hymenoptera stings.

To our knowledge, the presented data are the first to apply modern dimension reduction and clustering methods to the field of Hymenoptera venom allergies and show a diagnostic benefit. We believe that complex interactions in IgE-mediated allergies are reflected in different specific IgE sensitization patterns, which cannot be assessed by classic, linear statistics. In line with these findings, other fields in allergology related to IgE-mediated type I allergies, like asthmatic diseases, showed the benefit of a wholesome analysis of more than a single allergen. We present evidence that there is no need for individual cutoff-values for every single specific IgE antibody in Hymenoptera venom allergies, as the raw quantification of specific IgE antibodies can be used in clustering algorithms to subsequently derive meaningful clinical implications.

In other fields, the discovery of new recombinant allergens improved diagnosis and risk stratification based on an individual single allergen sensitization. Advances have been shown for recombinant allergens like rPru p 3^43^^,^^44^ in peach allergies and rAra h 1, 2, 3 in peanut allergies.^45^^,^^46^ Milk and egg allergies,^17^ allergic rhinitis, and asthma have also profited from new recombinants.^47^ In Hymenoptera venom allergies, CRD increased the sensitivity for sensibility detection.^48^, ^49^, ^50^ Particularly rApi m 10 was shown to be a risk factor for treatment failure of honey bee venom allergies^51^ and an indicator for a primary sensitization to honey bee venoms.^52^ Other Hymenoptera recombinants showed only questionable benefit for allergy diagnostics.^33^^,^^53^ We demonstrated that the usefulness of rApi m 10 can be extended as a quantitative input in a wholesome assessment with other allergens using dimensionality reduction and clustering methods. Similar behavior was observed for peach allergies, where different co-sensitizations between rPru p 3, p 1 and p 4 potentially enhanced risk assessment.^44^

In line with the previous studies, not all recombinants appear to be of equal value in Hymenoptera venom allergies. While rApi m 10 was a good single-allergen predictor as previously known, all other recombinants (rApi m 1, 2, 3, 5, rVes v 1 and 5) did not provide a clear benefit for clinical interpretation. While Api m10 stands out in bee allergy significance, we still must consider the other recombinants at lower levels, as low-titer sensitizations can also cause severe clinical reactions^54^ and indicate the need for immunotherapy. Sole reliance on predominant allergens like Api m10 could be misleading.

True dual sensitization to both bee and wasp venom differs significantly from mere cross-reactivity, such as specific IgE against CCDs without elevated anaphylactic reaction risk. Cross-reactivity between venoms often results from shared allergenic components, like CCDs. In contrast, true dual sensitization implies independent sensitizations to each insect's distinct allergens. This differentiation is crucial for diagnosis, management, and therapeutic strategies, including double venom immunotherapies. Certain studies suggest the basophil activation test for differentiating true dual sensitization.^55^

The abundance of available specific IgE recombinants in Hymenoptera venom allergies necessitates responsible and economical use. As the previous results suggest, redundancy and clinically irrelevant sensitizations exist when testing the whole Hymenoptera panel. We attempted to replicate the results with a more cost-efficient use of the available recombinants (honey bee [i 1], wasp [i 3], rApi m 10, and rVes v 1). We were able to retain most clustering characteristics with only 40% of the allergens.

Our study has several limitations that must be acknowledged. First, due to the constrained sample size, our findings are primarily based on internal validation, with external testing and predictions not conducted. Second, the specificity of our cohort — comprising largely of hunters and fishers — restricts the general applicability of our results. This group has a heightened exposure to Hymenoptera stings, which might not mirror the broader population's experience. Third, the dendrogram-based clustering, while insightful, is not without challenges; it is not a fully automated method and demands manual parameter calibration by researchers. It does offer the advantage of not requiring pre-set cluster numbers, unlike some other clustering methodologies. Despite these challenges, our study offers valuable perspectives into Hymenoptera venom allergies, aiding risk assessment and suggesting ways to enhance the efficiency of in-vitro specific IgE testing.

## Abbreviations

CCDs, cross-reactive carbohydrate determinants; CRD, component-resolved diagnosis; HDBSCAN, Hierarchical Density-Based Spatial Clustering of Applications with Noise; UMAP, Uniform Manifold Approximation and Projection.

## Funding

Parts of this study were supported by an unrestricted research grant provided by Thermo Fischer Diagnostics GmbH, Freiburg, Germany.

## Availability of data and materials

The datasets used and/or analyzed during the current study are available from the corresponding author on reasonable request.

## Author contributions

Robert Kaczmarczyk (RK), Alexander Zink (AZ), and Tobias Lasser (TL) were involved in the conceptualization and methodology of the study. RK curated the data and performed the formal analysis. TL, AZ, and RK were responsible for project administration, software development, and supervision. AZ provided resources for the study. Validation was carried out by RK, AZ, and TB. RK created the visualizations for the manuscript. The writing process involved Johannes Ring (JR), Tilo Biedermann (TB), TL, RK, and AZ, with RK, AZ, and TL preparing the original draft and Johannes Ring (JR) and Tilo Biedermann (TB) taking part in the review and editing process. All five authors (RK, AZ, TL, TB, JR) have directly accessed and verified the underlying data reported in the manuscript.

## Ethics approval and consent to participate

The study was reviewed and approved by the local ethics committee of the Medical Faculty of the Technical University of Munich under the reference number 604/19 S.

## Authors’ consent for publication

All authors approve this manuscript to be submitted to the World Allergy Organization Journal.

## Declaration of competing interest

AZ has received speaker's honoraris from Alk-Abelló and unrestricted research grants from Alk-Abelló and Phadia-Thermo Fisher.

TB gave advice to or got an honorarium for talks or research grant from the following companies: AbbVie, Alk-Abelló, Celgene-BMS, Lilly Deutschland GmbH, Mylan, Novartis, Phadia-Thermo Fisher, Sanofi-Genzyme, Regeneron, Viatris.

JR gave advice to or got an honorarium for talks or research grant from the following companies: AbbVie, Allergika, Leo, L'oreal, Alk-Abelló and Mylan-Viatris.

All other authors have no conflict of interest within the scope of the submitted work.
